# PHI-base: the pathogen–host interactions database

**DOI:** 10.1093/nar/gkz904

**Published:** 2019-11-16

**Authors:** Martin Urban, Alayne Cuzick, James Seager, Valerie Wood, Kim Rutherford, Shilpa Yagwakote Venkatesh, Nishadi De Silva, Manuel Carbajo Martinez, Helder Pedro, Andy D Yates, Keywan Hassani-Pak, Kim E Hammond-Kosack

**Affiliations:** 1 Department of Biointeractions and Crop Protection, Rothamsted Research, Harpenden AL5 2JQ, UK; 2 Cambridge Systems Biology Centre and Department of Biochemistry, University of Cambridge, Cambridge CB2 1GA, UK; 3 Molecular Connections, Kandala Mansions, Kariappa Road, Basavanagudi, Bengaluru 560 004, India; 4 European Molecular Biology Laboratory, European Bioinformatics Institute, Wellcome Genome Campus, Hinxton, Cambridge CB10 1SD, UK; 5 Department of Computational and Analytical Sciences, Rothamsted Research, Harpenden AL5 2JQ, UK

## Abstract

The pathogen–host interactions database (PHI-base) is available at www.phi-base.org. PHI-base contains expertly curated molecular and biological information on genes proven to affect the outcome of pathogen–host interactions reported in peer reviewed research articles. PHI-base also curates literature describing specific gene alterations that did not affect the disease interaction phenotype, in order to provide complete datasets for comparative purposes. Viruses are not included, due to their extensive coverage in other databases. In this article, we describe the increased data content of PHI-base, plus new database features and further integration with complementary databases. The release of PHI-base version 4.8 (September 2019) contains 3454 manually curated references, and provides information on 6780 genes from 268 pathogens, tested on 210 hosts in 13,801 interactions. Prokaryotic and eukaryotic pathogens are represented in almost equal numbers. Host species consist of approximately 60% plants (split 50:50 between cereal and non-cereal plants), and 40% other species of medical and/or environmental importance. The information available on pathogen effectors has risen by more than a third, and the entries for pathogens that infect crop species of global importance has dramatically increased in this release. We also briefly describe the future direction of the PHI-base project, and some existing problems with the PHI-base curation process.

## INTRODUCTION

Infectious diseases have a profound influence on every aspect of society. Diseases are a major concern to plant, animal, human, and ecosystem health. Globally infectious diseases threaten food security, human community structures, and the biodiversity of natural ecosystems (1–3). The increasing effects of climate change, human migration, and the globalisation of the trading of fresh goods have resulted in a rise in the incidence and severity of existing disease problems, as well as the emergence of a cohort of novel pathogen species and zoonoses (4). In addition, the (re)acquisition of resistance to anti-infective chemistries—coupled with a rise in legislation banning or restricting existing chemistries—means the burden of microbial infections is of ever growing concern to human, animal and plant welfare (5,6). In the United Kingdom alone, the total economic burden from infectious diseases is estimated at £30 billion annually, and accounts for 7% of all deaths (7).

Infectious diseases are a consequence of complex and dynamic interactions between pathogen virulence factors, and host cell recognition and response systems (8–10). It is increasingly clear that studying these interactions across the tree of life is a fertile ground for uncovering crucial biological principles that control the interaction outcome. In addition, in the post-genomics era—with the ever-decreasing costs for whole genome sequencing, genome assembly, and gene prediction—there is intense scientific and commercial interest in comparative pathogen genomics, as well as whole genome protein–protein interaction predictions and comparisons to identify functionally homologous genes, and to pinpoint species-unique genes and pathways. This increased understanding of the dynamics of a wide range of interactions contributes to the two predominant approaches available for combating infectious disease: namely, stimulating the host immune system to prevent infections, and minimizing the use of chemicals to eliminate infectious agents (11–13).

The pathogen–host interactions database (PHI-base) was established in 2005 and is freely available at www.phi-base.org. PHI-base contains expertly curated molecular and biological information on genes proven to affect the phenotypic outcome of pathogen–host interactions (14,15). All PHI-base entries are supported by strong experimental evidence from a peer reviewed publication. PHI-base catalogues experimentally verified pathogenicity, virulence, and effector genes from fungal, protist, and bacterial pathogens which infect plant, human, animal, and insect hosts. Genes tested but found not to affect the interaction outcome are also expertly curated. In PHI-base, the term ‘interaction’ is used to describe the observable function of one gene, on one host, on one tissue type (14). Nine high-level phenotypic outcome terms have been developed to permit the comparison of interactions across the entire tree of life (16). These terms are ‘loss of pathogenicity’, ‘reduced virulence’, ‘increased virulence’, ‘unaffected pathogenicity’, ‘effector’, ‘lethal’, ‘increased virulence (hypervirulence)’, ‘resistance to chemical’ and ‘sensitivity to chemical’. These high-level phenotypic outcome terms—although not yet supported by a formal controlled vocabulary—are particularly useful for bioinformaticians and biologists unfamiliar with the nuances of multiple pathogen–host interactions, but who wish to include pathogens with different lifestyles and host ranges in their comparative analyses. In addition, a PHIB-BLAST tool has been introduced to permit simple or advanced BLAST queries arising from functional genomics, transcriptomics, and proteomics experimentation.

In 2017, PHI-base joined the UK node of ELIXIR’s ‘Data for Life’ project as a gold-standard ‘agricultural omics data’ provider (17). PHI-base follows the FAIR data principles in order to make data findable, accessible, interoperable, and reusable (18). PHI-base also reuses data provided by external resources, including PubMed, NCBI taxonomy, UniProtKB, and the Gene Ontology (GO). A number of complementary multi-species databases on pathogens exist that also provide gene function annotation (recently reviewed by (14,19,20)). PHI-base is unique in describing a broad range of plant and animal pathogen–host interactions using the same controlled vocabulary consistently across >250 species.

In this article, we report on a major increase in PHI-base gene content, new database features, integration with complementary databases, and our immediate plans using new funding.

## RESULTS AND DISCUSSION

### Biological data

Version 4.8 of PHI-base (released in September 2019 and described in this article), contains data on 6780 genes, 13801 interactions, 268 pathogens, 210 hosts and 3454 references. This version includes 71% more interactions, each annotated with a phenotype, compared to PHI-base version 4.2 described in (14). Bacteria and fungal pathogens represent the majority of the interaction data, with a near 50:50 split of entries; whilst protists, nematodes and insects represent 3.6% of the species (Table 1). The fungal pathogen interactions are dominated by the Ascomycetes, which covers 88.5% of annotated fungal interactions (5929 interactions, 100 species); this is followed by the Basidiomycetes, which only cover 11.4% of annotated interactions (762 interactions, 11 species). In total, 5755 phenotype interactions describing experimental data on 2320 genes from 1235 newly curated publications are included up to March 2019.

**Table 1.** Summary of pathogen groups, interactions and phenotypes within PHI-base version 4.8

Phenotype/pathogen	Bacterium	Fungus	Protist	Nematode	Insect	Totals
Number of pathogens	131	112	17	6	2	268
Interactions in total	6608	6696	463	24	10	13801
Loss of pathogenicity	204	696	7	1	0	908
Reduced virulence	3054	2960	96	13	0	6123
Unaffected pathogenicity	1375	2202	60	0	0	3637
Effector (plant avirulence determinant)	1511	468	263	9	10	2261
Increased virulence (hypervirulence)	433	173	28	1	0	635
Lethal	18	156	9	0	0	183
Chemical target: resistance to chemical	7	29	0	0	0	36
Chemical target: sensitivity to chemical	6	8	0	0	0	14
Enhanced antagonism	0	4	0	0	0	4

The number of pathogenic species in PHI-base was capped at 268 and includes a small number of newly emerging pathogens under intense investigation. Plant infecting pathogens—namely bacteria, fungi, protists, nematodes and insects—represent 60% of the species in PHI-base (Table 2). Amongst these, there is an almost equal split between cereal and non-cereal infecting species. Woody tree infecting species provide 1004 interaction entries (7.3% of plant pathogen interactions). Amongst the 32 human and animal infecting pathogens, an increasing number are now being tested on non-vertebrate species: for example, various insects, nematodes and crustaceans. These non-vertebrate pathogen interactions now account for 23% of database entries (Table 2).

**Table 2.** Summary of the number of host species and interactions within PHI-base version 4.8

Phenotype	Plant	Vertebrate	Insect	Nematode	Others
Host species	131	32	24	1	22
Interactions in total	8248	4439	696	258	81
Loss of pathogenicity	650	233	15	9	1
Reduced virulence	2885	2655	386	137	60
Unaffected pathogenicity	2326	1004	193	98	16
Effector (plant avirulence determinant)	2001	233	23	1	3
Increased virulence (hypervirulence)	244	300	77	13	1
Lethal	101	80	2	0	0
Chemical target: resistance to chemical	27	3	0	0	0
Chemical target: sensitivity to chemical	13	1	0	0	0
Enhanced antagonism	4	0	0	0	0

As in previous versions of PHI-base, the highest number of pathogen–host interactions tested in molecular genetic studies and reported in the literature are from the filamentous fungal pathogens *Fusarium graminearum* and *Magnaporthe oryzae*, which cause various diseases on staple crops, such as wheat, rice and maize (Table 3). The most highly represented plant-infecting bacteria are *Ralstonia solanacearum*, a pathogen of potato and other Solanaceae crops; and *Xanthomonas oryzae*, a pathogen of rice. For the animal kingdom, the most frequently studied pathogens include the human pathogens *Salmonella enterica*, *Candida albicans* and *Pseudomonas aeruginosa* (Table 3). Amongst the top 30 species present in PHI-base, phenotypic interaction information—from single, double and occasionally multiple gene deletions—is provided for each species: from a minimum of 32 genes to a maximum of 1340 genes. However, for the cereal infecting fungus *Pyrenophora tritici-repentis*, only five genes have been explored over 142 interactions. Overall, the 30 top species in PHI-base consist of 12 fungi, 1 protist and 17 bacteria, and together these covers 71% of total interactions and 88% of total genes.

**Table 3.** Top species and interactions within PHI-base version 4.8

Pathogen	Interactions	Genes	Loss of pathogenicity	Reduced virulence	Increased virulence	Effector	Unaffected pathogenicity	Lethal	No. of tested host species
*Fusarium graminearum*	1571	1340	36	516	8	0	917	94	13
*Magnaporthe oryzae*	1273	738	279	501	10	84	398	1	7
*Ralstonia solanacearum*	666	132	16	43	0	597	9	1	9
*Salmonella enterica*	664	412	8	381	45	108	122	0	11
*Xanthomonas oryzae*	512	224	3	96	24	306	83	0	3
*Erwinia amylovora*	450	135	34	165	55	15	181	0	5
*Candida albicans*	448	343	48	305	11	0	80	4	12
Pseudomonas aeruginosa*	440	220	19	218	34	4	165	0	16
*Botrytis cinerea*	368	147	24	205	10	4	123	0	26
*Ustilago maydis*	360	264	48	187	8	17	100	0	3
*Aspergillus fumigatus*	309	207	30	128	14	0	93	42	4
*Cryptococcus neoformans*	305	203	44	184	17	0	50	10	8
*Pseudomonas syringae*	293	170	1	53	9	191	38	1	13
*Escherichia coli*	264	169	1	167	15	11	69	1	13
*Staphylococcus aureus*	212	139	12	137	22	2	38	1	10
Fusarium oxysporum*	209	131	24	90	8	27	60	0	17
*Xanthamonas campestris*	180	110	11	95	4	0	39	2	8
*Klebsiella pneumoniae*	173	134	4	72	4	0	93	0	4
*Streptococcus pneumoniae*	152	106	2	110	4	0	30	6	5
*Mycobacterium tuberculosis*	150	112	3	64	36	1	46	0	4
*Candida glabrata*	148	43	0	89	6	0	52	1	3
*Verticillium dahliae*	145	60	14	64	9	24	34	0	16
*Listeria monocytogenes*	142	69	2	102	17	3	18	0	10
*Pyrenophora tritici-repentis*	142	5	0	3	1	138	0	0	3
*Enterococcus faecalis*	132	32	1	82	3	0	46	0	5
*Hyaloperonospora arabidopsidis*	127	70	0	1	3	123	0	0	5
*Streptococcus pyogenes*	121	68	0	67	19	0	33	2	7
*Xanthomonas citri*	119	33	6	18	3	88	4	0	6
*Vibrio cholerae*	117	71	1	83	2	0	31	0	6
*Beauveria bassiana*	108	84	4	70	8	2	24	0	11
**TOTALS**	9835	5971	675	4296	409	1745	2976	166	

*Pathogen species able to infect both plant and animal hosts.

Since 2015, there has been an emphasis on increasing the curation of pathogen gene modifications that result in a hypervirulence phenotype on the host. This has steadily risen from 112 genes (version 3.8) to 233 genes (tested in 324 interactions) (version 4.2), to 475 genes (tested in 635 interactions) (ver. 4.8). Hypervirulence phenotype interactions now account for 4.6% of all database entries and are particularly prevalent amongst bacterial pathogen entries (Table 1). This increasing number of hypervirulent interactions indicates that many additional aspects of the negative regulation of key pathogenicity processes—occurring during infection and colonization of both plant and animal hosts—have been identified. This gene set continues to warrant close monitoring in pathogen populations when attempting to explore, and then mitigate, the emergence and spread of hypervirulent pathogens associated with severe disease outbreaks (21).

A second major curation effort for PHI-base has been to increase coverage of pathogen effectors (14). An effector is an entity derived from a pathogenic or non-pathogenic species, that either activates or suppresses host defences or other host responses. Interactions involving effectors have risen by 35%: from 1668 (version 4.2) to 2261 (version 4.8). This category now represents 16% of the dataset, with data derived from 83 species, mostly plant pathogens (Table 4). In total, 67% of the effector entries (1511 interactions) are from bacterial species; there is also a considerable number of entries from five obligate fungal rust or powdery mildew species, and one obligate protist species (*Hyaloperonospora arabidopsidis*). Based on data curated in PHI-base, the experimental method of choice for studying effector function is evaluating transient expression in a host or non-host species: transient expression tests account for 573 interactions across 28 pathogen species.

**Table 4.** Summary of the pathogenic species providing the most information on effectors

Pathogen - 83 species	Interactions
**Bacteria - 40 species**	**1511**
*Ralstonia solanacearum*	597
*Xanthamonas oryzae*	306
*Pseudomonas syringae*	191
*Salmonella enterica*	122
*Xanthamonas citri*	88
**Fungus - 25 species**	**471**
*Pyrenophora tritici-repentis*	138
*Magnaporthe oryzae*	84
*Passalora fulva*	57
*Fusarium oxysporum*	27
*Ustilago maydis*	17
**Obligate fungal biotrophs - 5 species**	**65**
*Melampsora* species	33
*Puccinia* species	27
*Blumeria* species	5
**Protist / 10 species**	**263**
*Hyaloperonospora arabidopsidis*	123
*Phytophthora sojae*	51
*Phytophthora capsici*	38
*Phytophthora infestans*	29
**Nematodes and insects - 3 species**	**10**

In 2015, nine high level phenotypic terms were introduced to the curation process, to permit researchers to explore the database across a wide range of taxonomically diverse species which exhibit varied pathogenic lifestyles (16). The phenotype term ‘reduced virulence’ is the most highly represented and applies to 44% of database entries. The second most frequent term is ‘unaffected pathogenicity’, at 26%. The majority of the ‘unaffected pathogenicity’ phenotypes have been reported for plant pathogens (64%), however an increasing number (1004) are from animal pathogens (compared to 80 interactions in version 3.6, and 280 interactions in version 4.2). This change appears to have arisen primarily because, within an individual publication, the number of host species tested, or the number of pathogen genes tested has increased; also, comparative results may be included from single, double, and multiple-gene deletion mutants. The number of articles reporting entirely negative data remains small. These negative outcomes are usually presumed (by the respective authors) to indicate that the gene product does not have a functional role in the pathogenic process under investigation, or that gene redundancy exists.

The high-level phenotypes for all interactions are summarized in Table 1 (for pathogen species) and Table 2 (for host species). A total of 183 PHI-base entries have been assigned the ‘lethal’ phenotype, consisting of 7 plant-infecting pathogens, 12 animal-infecting pathogens and 1 insect-infecting pathogen. The majority of lethal phenotype annotations are for fungal species, in particular *Fusarium graminearum* (94 entries), for which genome-wide single gene replacement studies have been completed for all predicted transcription associated proteins (22), the predicted protein kinases (the kinome) (23), protein phosphatases (the phosphatome) (24), and—most recently—the predicted plasma membrane spanning G-protein coupled receptors (25,26). In these large-scale experiments, no transformants were recovered in repeat experiments, whilst transformants were recovered for many other genes. Thus, the authors considered that the gene's function was ‘essential for life’. The human pathogen *Aspergillus fumigatus* has also contributed a disproportionately high number of lethal phenotype entries, with 42 of the 207 genes tested (20%) falling into this category where a targeted screen for essential genes has been initiated (27). However, amongst the 30 species with the most interactions in PHI-base (Table 3), 17 species have no ‘lethal’ category entries, whilst a further 8 species only provide 1 or 2 lethal entries.

An increasing number of interactions involving human and animal pathogens are now being tested in non-vertebrate species (Table 2). In these bioassays, a wide range of insect larvae are used, including: *Galleria mellonella* (greater wax moth), *Plutella xylostella* (diamondback moth), and *Bombyx mori* (domestic silkworm); as well as adult insects, specifically *Drosophila melanogaster* (fruit fly). Other studies have used the nematode *Caenorhabditis elegans* (roundworm), the slime mold *Dictyostelium discoideum*, the free-living amoeba *Acanthamoeba castellanii*, or various crustaceans: such as shrimp species from the genus *Artemia* and *Penaeus*; and bivalve species, such as oysters from the genus *Crassostrea*. The increasing adoption of the 3Rs principle (replacement, reduction, and refinement) in place of animal models is the main contributing factor to the rising number of non-vertebrate entries (28).

With increasing concerns over global food security, researchers in the international community are being encouraged to investigate host plant-pathogen interactions in crop species, rather than just model pathosystems (29). In addition, the availability of the published completed reference genome for hexaploid bread wheat (*Triticum aestivum*) from the International Wheat Genome Sequencing Consortium (RefSeq v1.0) (https://www.wheatgenome.org/) (30) is increasing the pace of discovery for many wheat infecting species. Table 5 shows the interaction entries involving major food and feed crops: namely wheat, rice, maize, barley, tomato, potato, and *Brassica*. Together, these seven host plant species provide 37% of the data in PHI-base (5096 interactions) and involve 79 pathogenic species (60% of plant pathogen species in PHI-base). In contrast, the three model species *Arabidopsis thaliana*, *Nicotiana benthamiana*, and *Nicotiana tabacum* provide only 5% of the data (688 interactions). The high number of 48 pathogenic species tested using *N. benthamiana* and *N. tabacum* is predominantly due to the availability of *Agrobacterium*-mediated transient expression assays to test the function of effector proteins.

**Table 5.** Crop plant and model plant species contributions to PHI-base version 4.8

Host plant	Interaction entries	No of pathogen species	Loss of pathogenicity	Reduced virulence	Increased virulence	Effector	Unaffected pathogenicity
**Crop species**							
Wheat	1790	18	71	513	15	149	923
Rice	1371	9	172	464	33	366	324
Maize	661	13	66	381	19	18	176
Barley	463	9	95	163	2	33	169
Tomato	590	30	56	190	9	195	139
Potato	112	15	1	59	8	20	24
*Brassica*	109	12	16	55	3	15	20
**Model species**							
Arabidopsis	359	28	7	97	13	198	44
Tobacco (*N. benthamiana* and *N.tabacum*)	329	47	7	71	21	202	28
**TOTALS (8 crop species)**	5784	102 (different species)	491	1993	123	1196	1847

### Mapping PHI-base phenotypes to Ensembl Genomes and FungiDB

PHI-base supplies phenotypic annotation for over 100 crop-plant-infecting microbial pathogens into Ensembl Genomes (31). This contribution was initiated as part of the PhytoPath project (32). Recently, the implementation of an improved mapping pipeline developed by Ensembl has contributed to an increase in the number of genomes with PHI-base annotations by a factor of 8.7 in the total genomes of bacteria, fungi and protists compared to 2017 (De Silva *et al.*, NAR Database issue 2020, submitted). Also, as a result of extrapolating annotations for conserved genes to closely related species, Ensembl have now applied PHI-base annotations to over 14 000 genes in over 1000 genomes. These can provide potential clues for experimental validation in other pathogens. Phenotype annotations are also provided to FungiDB (33). FungiDB release 46 (October 2019) integrates 2633 PHI-base annotations, mapping to 1636 genes for 18 FungiDB hosted genomes. In addition to pathogen–host interaction annotations, several *in-vitro* phenotypes including growth, sporulation and penetration defects are displayed.

### Migration to reference sequence UniProt IDs

PHI-base provides links to UniProt IDs when these accessions exist in UniProt Knowledgebase (34). These links can provide further molecular protein annotation, including GO terms. However, new genomes are sequenced, and existing genomes are re-annotated. This can generate multiple gene IDs and protein IDs for the same gene, causing interoperability issues. We are currently migrating to a system where we consistently use the UniProt identifier from the reference strain as listed by UniProt, rather than IDs from alternative (non-reference) strains. PHI-base has over 15 years of curated literature, and therefore contains ∼11% legacy genes with no link to UniProt; here in most cases GenBank and EMBL records are referenced. For the genes originally curated with Uniprot IDs, ∼10% were in the meantime moved to the UniParc sequence archive. Thus, a challenge exists to frequently review and update PHI-base records, until microbial pathogen proteomes become sufficiently refined and available at UniProtKB. In the meantime, single-species community-based efforts, such as FusariumMutantDB (https://scabusa.org/FgMutantDb) (35), can effectively support PHI-base by providing mapping files for legacy gene IDs in several genome assemblies/strains to reference strain IDs available at UniProtKB. BLAST mapping of PHI-base proteins using Blast2GO software (Vers. 5.2.5) (36) using default parameters against the UniProtKB/TrEMBL (release2019_07) identified 937 sequences without GO associations. These sequences include many fungal species-specific effectors, for which currently GO terms are being created.

### PHI-base BLAST tool

PHI-base has a strong focus on providing curated phenotype data, with less emphasis on providing bioinformatics tools. Excellent tools for genome browsing and sequence investigations are provided for example by Ensembl Genomes, FungiDB and other genomic resource providers (33,37). However, since 2017 we have provided an online sequence-to-phenotype BLAST search tool, called PHIB-BLAST. This allows users to map their own sequences to PHI-base accessions and the reported phenotypic outcomes are displayed in the BLAST result header, to give immediate comparisons between species. Additionally, this information is also made available for download in FASTA format, where PHI-base information is embedded in the single-line FASTA header for each protein sequence.

### PHI-base usage

All of the publications citing PHI-base use are cited in the ‘about’ section of the database. Currently, 367 articles have cited PHI-base and 60% of these have been published in the past 5 years. New research investigations using PHI-base information cover multiple active fields of research, including gut microbiomes, effector discovery, diagnostic markers for the early ‘in host’ detection of pathogens and finding lethal phenotypes in human pathogens to aid the drug discovery process. For those wishing to query past versions of PHI-base, these have been made available on our ‘data’ repository on GitHub (https://github.com/PHI-base). PHI-base is accessed by users in 130 countries over six continents. Over the past 3 years PHI-base usage has remained relatively stable at between 9000–16 000 searches and >400–600 full downloads per annum.

### Outreach to inspire the next generation

To help PHI-base reach a different audience, a STEM (Science, Technology, Engineering and Mathematics) outreach article was recently published highlighting the importance of big data, bioinformatics and plant pathology (https://futurumcareers.com/saving-plants-from-disease). This article was aimed at an audience of 11–19-year olds to inform and enable them to consider career options within these fields. Example case studies were taken from PHI-base and PhytopathDB. Accompanying worksheets (https://futurumcareers.com/Kim_Hammond-Kosack-activity-sheet.pdf) were provided to stimulate discussions and ideas within classrooms and beyond.

### Future directions

#### PHI-Canto and ontologies

As reported previously (14) we have developed the multi-species web-based curation tool PHI-Canto (canto.phi-base.org). PHI-Canto is an implementation of the Canto community curation tool, developed and used by the fission yeast database PomBase (38). In addition to supporting professional biocurators, researchers will be able to directly contribute annotations from their publications to PHI-base. PHI-Canto supports the annotation of GO, phenotypes, modifications and interactions.

Curation in PHI-Canto involves specifying a publication (using a PubMed ID), entering experimental pathogen and host genes (using UniProtKB IDs), creating genotypes (by listing alleles), annotating genotypes with one or more PHIPO (pathogen host interactions phenotype ontology) terms, and selecting an experimental evidence code. Pathogen–host interaction phenotypes are connected to the underlying genotypes of both the pathogen and the host (multi-species genotypes). Physical protein interactions—such as those identified in yeast two-hybrid or co-immunoprecipitation experiments—can also be curated which will be particularly useful in recording the direct interacting host targets of pathogen effectors.

Recent developments to PHI-Canto include three main improvements. First, the handling of host genes and genotypes. Second, the ability to capture increasingly complex pathogen host interactions, involving incremental changes to either, or both, the pathogen and the host. Third, mechanisms to capture single species phenotypes for the pathogen and the host. PHIPO was recently registered at the Open Biological and Biomedical Ontology (OBO) Foundry (http://www.obofoundry.org/ontology/phipo.html) to promote reuse in the pathogen community.

PHI-Canto will enable accurate biocuration of pathogen–host interaction data into PHI-base by the international community. With increased PHI-Canto use this will ensure PHI-base can keep up-to-date with the ever-growing number of publications and newly developed experimental techniques. Researchers interested in trialling PHI-Canto are encouraged to contact us by email (curation@phi-base.org).

#### Improving strain identification and disease curation

Curating strain and disease names are problematic because a wide range of synonyms exist that are inconsistently used and published in different research communities. We have developed a standardized list of strains of importance to PHI-base and currently continue to revise inconsistencies in legacy data. A list of infectious disease names from PHI-base is currently being standardized to a set of external disease ontologies for animals and plants. The revised nomenclature will be used in future PHI-base releases and within PHI-Canto.

#### Curation of the fungicide and anti-infective literature

PHI-base is curating publications describing the target sites of some anti-infective chemistries although this has not been a high priority (Table 1). We plan to increase the coverage of fungicide and anti-infective literature over the next 2 years. To support this work, we have curated a list of anti-infective agents, including a description of the anti-infectives’ function, and cross-references to other databases (FRAC codes, CHEBI IDs, and CAS numbers) where available. This inventory is available from our ‘data’ repository on Github (https://github.com/PHI-base). A pilot text mining study with Molecular Connections (PHI-base curation partner), will test bespoke machine learning algorithms using the anti-infectives list to identify additional papers containing potential fungicide and other anti-infective targets for future curation.

#### Access to PHI-base annotations in graphical displays of biological networks

KnetMiner (https://knetminer.org) is a digital research assistant with a Google-like search interface, predictive graph algorithms and interactive features to visualize biological knowledge networks (39,40). KnetMiner mines millions of relations in a genome-scale knowledge network to identify novel clues about genes, gene networks, and diseases (41,42). KnetMiner can search an integrated database of crop and model organism genomes, curated databases such as PHI-base, gene expression, gene interaction information, ontologies and the scientific literature to produce a ranked answer with evidence codes within seconds. The user can then interactively explore the auto generated knowledge network, hiding noisy or untrustworthy relations. So far knowledge networks containing PHI-base data have been developed for the cereal infecting fungal pathogens *Fusarium graminearum* and *Zymoseptoria tritici*. For plant and crop species, available networks include Arabidopsis, wheat and rice. In the future, our plans are to link knowledge networks for PHI-genes from Knetminer directly into PHI-base.

#### PHI-annotations into additional databases

PHI-base currently provides phenotype data to a variety of resources, including Ensembl Genomes (Protists, Bacteria and Fungi), FungiDB, Knetminer, FusariumMutantDB and GLOBI (43). Future plans include linking out to Ensembl Plants and to the thousands of fungal genomes and hundreds of protist genomes in the MycoCosm database provided by the Joint Genome Institute (JGI) (44). One of the advantages of MycoCosm is that the genomes of pathogenic and non-pathogenic species are displayed and queried via the use of a navigation tree which assists users with minimal knowledge of taxonomic relationships and groups.

We will supply Gene Ontology annotations to GO, from where they will be distributed to other resources, including UniProtKB. PHI-base curators are working closely with the manual curation team of UniProtKB/Swiss-Prot to ensure that gene names and strains are consistent between entries, and we will explore mechanisms to share phenotype annotations with UniProtKB.

## DATA AVAILABILITY

1. PHI-base 4: www.phi-base.org
2. PHI-base GitHub page: https://github.com/PHI-base
3. PHIB-BLAST: http://phi-blast.phi-base.org
4. PHI-wiki page: https://en.wikipedia.org/wiki/PHI-base
5. PHI-Canto (multi-species community annotation tool): https://canto.phi-base.org/
6. Linked resource - Ensembl genomes: http://ensemblgenomes.org
7. Linked resource - FungiDB: https://fungidb.org/fungidb/
8. Linked resource - Pombase: https://www.pombase.org/
9. Linked resource - KnetMiner: https://knetminer.org
10. Linked resource - ELIXIR UK: https://elixiruknode.org/
